# Effect of Psychosocial Interventions on Cancer's Caregiver Quality of Life: Meta-analysis

**DOI:** 10.2174/17450179-v19-e230927-2022-HT14-4336-1

**Published:** 2023-08-25

**Authors:** C Vasantha Kalyani, Kusum K. Rohilla, Pratima Gupta, Amit Gupta, Sweety Gupta

**Affiliations:** 1 College of Nursing, All India Institute of Medical Sciences, Deoghar, India; 2 Department of Microbiology, All India Institute of Medical Sciences, Deoghar, India; 3 Department of Surgery, All India Institute of Medical Sciences, Rishikesh, India; 4 Department of Radiation Oncology, All India Institute of Medical Sciences, Rishikesh, India

**Keywords:** Caregiver, Cancer, Psychological intervention program, Quality of life, Dementia

## Abstract

**Background:**

People living with cancer benefit greatly from informal caregivers. No previous meta-analysis was done to check the effect of psychological intervention on cancer caregiver's quality of life.

**Objectives:**

The goal of this meta-analysis was to check the effect of psychosocial interventions on Cancer's Caregiver quality of life and check the impact of various psychological intervention programs.

**Methods:**

A comprehensive literature search was conducted from January 2006 to April 2021 using PubMed, PubMed Central, Clinical Key, CINAHL Database, EBSCO, Google Scholar and Cochrane database.

**Results:**

The effect of psychological intervention programs on caregiver's quality of life was evaluated using a mean difference between experimental and control groups. A random-effects model was used to measure the mean difference (MD) for calculating the cancer caregiver's quality of life. The final report comprised eight trials with a total of 1142 participants. The caregiver intervention programme was found to improve cancer caregivers' quality of life, but not statistically significantly (mean difference=0.10; p<0.00001).

**Conclusion:**

According to this meta-analysis, The psychological intervention program positively affected cancer caregiver's quality of life. Further randomised controlled trials are required to prove that psychological interventional programs are successful strategies for improving cancer caregiver's quality of life.

## 1 INTRODUCTION

Advancements in technology and improvement in cancer diagnosis and care have resulted in a growing number of cancer survivors, as evidenced by data from International Agency for Research on Cancer (IARC). Approximately 17.0 million incidences of new cancer cases are reported annually; by 2020, more than 19.3 million new cancer cases are expected to be diagnosed and by 2040, predictions of up to 27.5 million new cancer cases globally [1, 2]. Approximately 43.5 million informal cancer caregivers globally; among them, one in every four caregivers spends 41 hours per week providing care to their adult or child cancer patients [3].

Informal caregivers are often family members, *i.e.* spouses, children or friends, who provide care to a person suffering from cancer, old age or dementia [4]. Around 70-80% of cancer treatment is provided by caregivers only, which are available to the patient throughout the disease process, *i.e.*, from diagnosis to death [5]. Caring for someone with cancer can be highly taxing; 50% of caregivers experience high levels of stress and depression [6], and 40% of caregivers suggest that they need assistance in handling their own physical stress and emotions [7, 8].

Caregiver's burden is described as the degree to which they negatively impact their physical, social, emotional, financial and spiritual well-being while caring for others [9]. Caregivers may face symptoms like depression, lack of social support, increased anxiety, loneliness, helplessness and fear of recurrence also [10]. A caregiver may face physical issues like exhaustion, poor sleep, weight gain or loss, and lack of exercise; all of these impact the caregiver's health status, *i.e.* decreased immune system function, coronary heart disease or early mortality [11]. Child or adult cancer patient caregivers may have musculoskeletal discomfort due to lifting and moving heavy weights or loads [12]. Thus all these factors affect the overall health status of cancer caregivers.

Furthermore, studies also show that a caregiver's physical, mental health and quality of life (QOL) is badly influenced while caring for cancer patients [13-15]. Burden on cancer caregivers will last for many years after the patient's initial cancer diagnosis until it decreases, which has evidence on their health and QOL consequences [16, 17]. As a result, the researchers developed various interventional programs for caregivers to mitigate physical and psychological stressors, automatically improving caregiver QOL [18]. This meta-analysis's main aim is to identify the effect of a psychological intervention program on a caregiver's quality of life and its impact on the caregiver.

## 2 MATERIALS AND METHODS

**Fig. (1):**
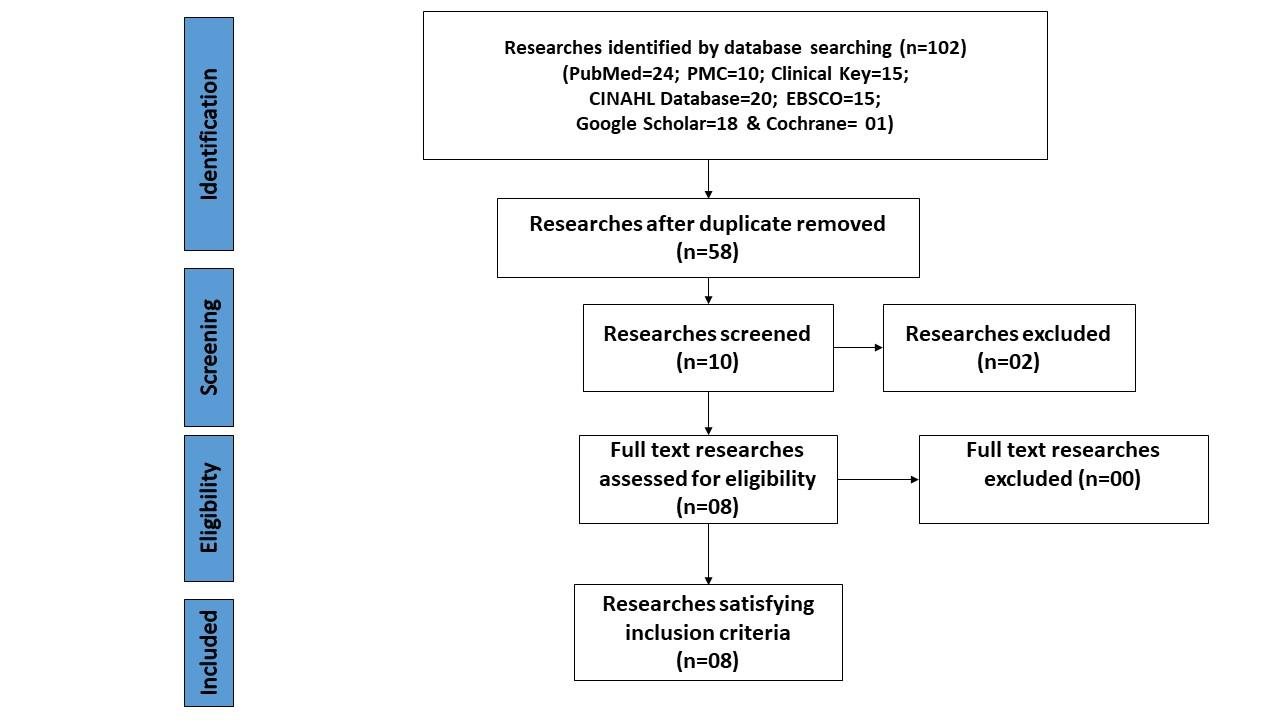
PRISMA guidelines-2020 for included studies [19].

By following PRISMA-2020 guidelines [19], this analysis protocol was written to identify the effect of a psychological intervention program on a caregiver's quality of life (Fig. **1**).

### 2.1 Source of Data and Study Selection Strategy

Randomised controlled trial (RCT) studies published online from PubMed, PubMed Central, Clinical Key, CINAHL Database, EBSCO, Google Scholar, and Cochrane database were included from January 2006 to April 2021. The quest was limited to peer-reviewed publications, papers written in English and research concerning human subjects. The search strategy for trials was done by following the PICO format, *i.e.* Population, Intervention, Control, and Outcomes. For our search trials, we use P= Caregiver, I=Cancer, C=Patients, and O=Quality of life. MeSH (Medical Subject Headings) terms used for the population were “Caregiver”, “Family caregiver”, “Spouse”, “Partner”, for intervention were “Psychological Intervention”, “caregiver intervention program”, “Cancer patients Intervention”, “Oncology caregiver intervention”, for control were “No Intervention”, “Control group” and for outcome were “Quality of life”, “QOL” terms were used to search database.

### 2.2 Selection of Study

Two reviewers (KKR and CVK) reviewed the study title, abstract and research design included by following reported guidelines for randomised controlled trials from January 2006 to April 2021. Trails related to caregivers providing care to adult cancer patients were included. Exclusion criteria included studies involving children cancer patients, and studies with qualitative methodology were excluded.

### 2.3 Outcome Measures

Both independent authors (KKR and CVK) looked at the primary outcomes of the included studies, such as study characteristics and participant bio-demographic profiles. The main goal is to evaluate the effect of psychological intervention programs on a caregiver’s quality of life.

### 2.4 Data Extraction

Data was extracted from the selected studies by two separate authors (KKR and CVK). Any differences in inclusion eligibility were resolved through discussion among the authors. Following the selection of each trial, the data was tabulated in the form of author name, year of publication, experimental and control group details, *i.e.* frequency, mean age, standard deviation and gender, experimental and control group intervention, research tools and study settings. Any necessary clarifications were obtained by emailing the correspondence author.

### 2.5 Risk of Bias and Quality Assessment

**Fig. (2):**
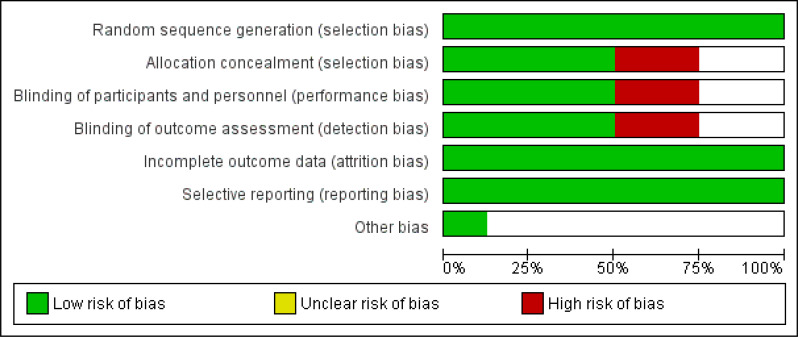
Graph of risk of bias.

**Fig. (3):**
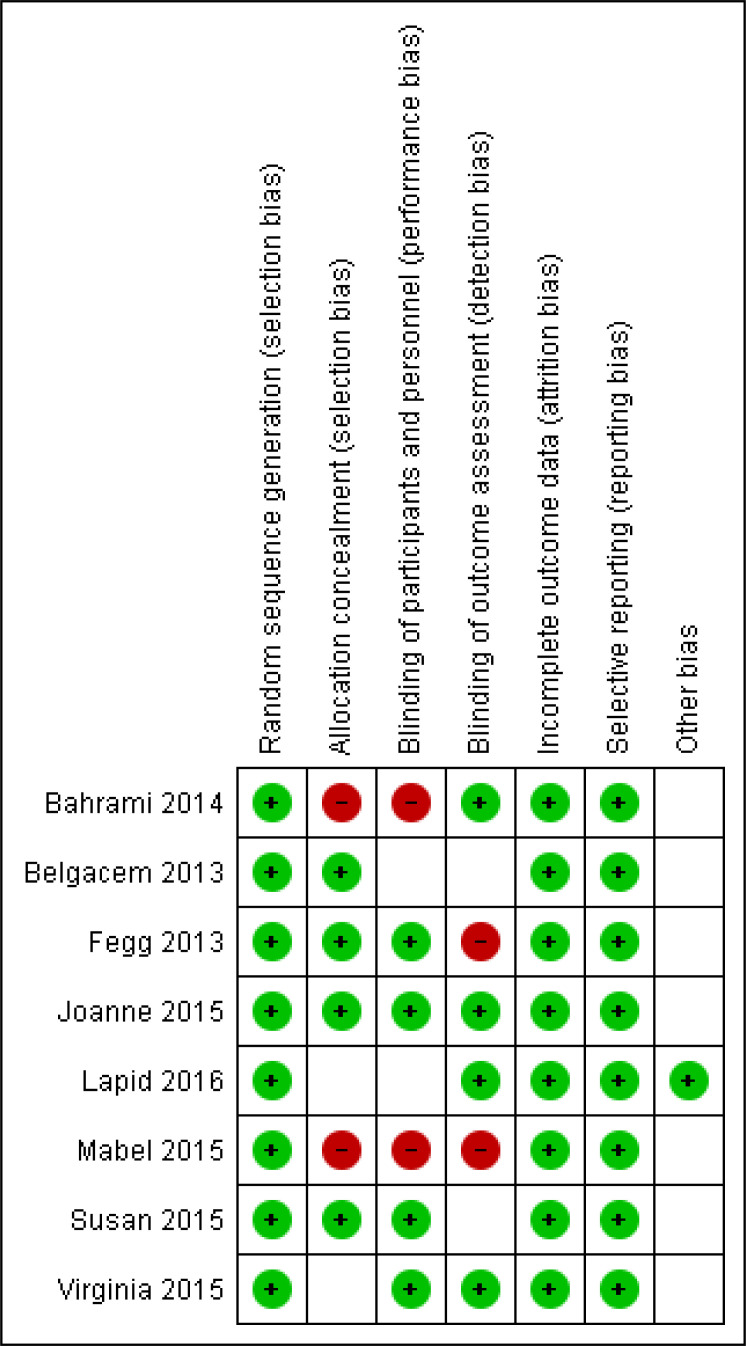
Summary of risk of bias for included studies.

The risk of bias was assessed separately by two authors (KKR and CVK) separately using Cochrane risk of bias guidelines [20]. Cochrane risk of bias graph mainly consists of six criteria, *i.e.* selection bias, performance bias, detection bias, attrition bias, reporting bias and other biases (Fig. **2**), and a risk of bias summary for each selected randomised controlled trial was obtained (Fig. **3**).

 

For each variable, all studies were categorised as low, high, or unclear risk. Disagreements between two independent authors (KKR and CVK) were settled by common consensus. Among eight selected trials, the Chi-square test was used to evaluate heterogeneity by using I^2^ statistics. Table **1** lists all of the data collected from trials included in the meta-analysis.

All eight trials that were included had adequately defined randomised sequence methods. Four trials (Belgacem 2013 [21], Fegg 2013 [22], Joanne 2015 [23] and Susan 2015 [24]) had properly defined allocation concealment, while two trials had an ambiguous status, and two trials had not done allocation concealment. Four trials (Fegg 2013 [22], Joanne 2015 [23], Susan 2015 [24] and Virginia 2015 [25]) listed a double-blinding design, while two trials were unclear, and two trials had not followed double-blinding design. Four research (Bahrami 2014 [26], Joanne 2015 [23], Lapid 2016 [27] and Virginia 2015 [25]) identified blinding of outcome assessors, while two trials had a high-risk bias, followed by three trials with unclear status. All eight trials had well-described outcome data and reporting bias. One trial (Lapid 2016 [27]) had reported other bias only (Table **1**) [28, 29].

### 2.6 Data Analysis

The final data analysis of the study was carried out using RevMan version 5.4, whose protocols are obtained from the current edition of the Cochrane Handbook [30].

Continuous data on caregivers' quality of life outcomes was presented as a mean difference (MD) with a 95% confidence interval. The funnel graph for the mean difference with the standard error was plotted to analyse the caregiver's quality of life to evaluate possible publication bias. The Chi-square test was used to evaluate heterogeneity using I2 statistics for selected trials. I^2^ values of 0% indicate no heterogeneity, 50% indicate minimal heterogeneity, and >50% indicate substantial heterogeneity, which was used to draw inferences from the data. For the final meta-analysis, the researchers used a random-effect model with a significant p =0.05 and an I^2^ statistic of 50%, suggesting heterogeneity.

## 3 RESULTS

### 3.1 Study Selection and Characteristics

Initially, the PRISMA-2020 guidelines [31, 32] were used to create the flow diagram that registered, screened, removed, and finally included in the search strategy. Using the PICO format (PubMed-24, PubMed Entral-10, Clinical Key-15, CINAHL Database-20, EBSCO-15, Google Scholar-18 and Cochrane-01), 68 studies were included, and 60 studies were omitted. As a result of the screening, eight full-text RCTs qualified, meeting all eligibility criteria of this metanalysis (Fig. **1**).

Table **1** shows the characteristics of the eight trials that were included. A total of 1142 people participated in the eight trials in which caregivers were given training programmes to help them sustain and maintain their quality of life. The primary outcome of cancer caregivers’ quality of life, all eight trials had complete results.

**Fig. (4):**
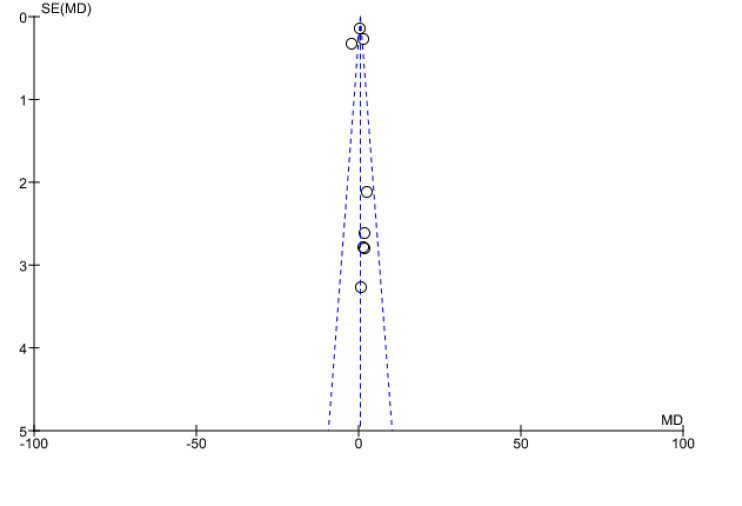
Funnel plot showing the quality of life of caregivers. (*SE (MD): Standard error (mean difference).

To assess the impact of psychological intervention programs on caregivers’ quality of life, funnel plots revealed no significant asymmetry from meta-analysis results. The funnel plot of the quality of life of cancer caregivers' mean difference revealed no signs of publication selection bias (Fig. **4**).

### 3.2 Primary Outcome: Effect of Caregiver Interventional Program on Cancer Caregiver’s Quality of Life

**Fig. (5):**
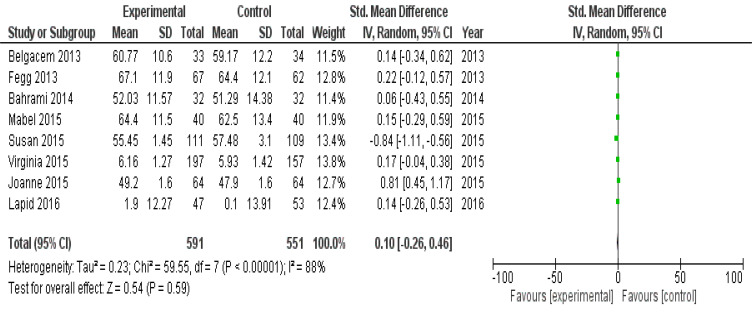
Forest plot showing the quality of life of cancer caregivers.

Total pooled results of eight studies (Fegg (2013) [22], Belgacem (2013) [21], Bahrami (2014) [26], Mabel (2015) [28], Susan (2015) [29], Joanne (2015) [23], Virginia (2015) [25] and Lapid (2016) [27] showed substantial difference in cancer caregiver quality of life with or without any intervention with mean difference of 0.10 [95% CI -0.26 to 0.46; p<0.00001]. The experimental group had 591 participants, while the control group had 551 participants. The pooled analysis revealed relatively high heterogeneity (I^2^=88%, p<0.00001) (Fig. **5**).

## 4 DISCUSSION

This present meta-analysis involved eight RCTs in which 1142 participants were overlooked to check the impact of various psychological intervention programs on caregivers’ quality of life and found that caregivers' quality of life improved. A systematic review reveals that cancer caregivers' quality of life improved and stress levels reduced by using various psychological intervention programs that focus on selfcare, interpersonal relationships, and symptom control of patients. [32] Another systematic review also appraises that implementing evidence-based practice for cancer caregivers improves their quality of life. This meta-analysis revealed that there is an urgent need to plan, perform, and report research on the impact of psychological intervention programs on caregivers' quality of life so that various psychological interventional programs can be planned and put for caregivers which mainly emphasis on efficacy and improvement of cancer caregivers’ quality of life [33].

The meta-analysis key finding also suggested that various psychological interventional programs for caregivers were not planned systematically to maximise the potential benefits of interventions to be adopted effectively. We can learn about the complexities of various operationalising implementation programs and their outcomes for cancer caregivers from an existing framework of various cancer centres. According to this meta-analysis, the documentation of cancer caregiver intervention research needs to be improved in order to promote their incorporation into practice. There seem to be two major concerns. To begin with, experiments were not planned in a way that maximised their chances of being implemented successfully. Second, in some cases, only a small amount of information is available that is applicable to the implementation of various interventional programs for caregivers.

Researchers also found that restrictions in reporting research, such as journal standards and their word counts, can restrict the ability to disclose evaluation data that includes implementation and relevant outcomes.

### 4.1 Limitations

This meta-analysis showed the effect of various caregiver intervention programs to improve their quality of life. Only randomised controlled trials are used in the present meta-analysis, in which the majority of confounding variables were excluded, which can influence any study results.

The researchers were unable to find caregiver interventional program enforcement or follow-up time in all included trials, which may have a direct impact on cancer caregivers’ quality of life.

## CONCLUSION

This meta-analysis found strong evidence that psychological intervention program has a positive impact on cancer caregivers' quality of life. More randomised controlled trials should be required to show the impact of psychological intervention programs and other strategies to improve cancer caregivers' quality of life.

## Figures and Tables

**Table 1:** Characteristics of included studies (n=8).

**Author & Year**	**EG/ CG**	**EG/ CG Age (SD)**	**EG/ CG Male & Female**	**EG**	**Research Tool**	**CG**	**Setting**
Fegg *et al.* [22] (2013)	67/62	54.3 (13.5)/ 54.7 (12.9)	M-17 (28%), F- 50 (72%)/ M-19 (23%), F- 43 (67%)	EBT (Existential Behaviour Therapy) treatment for six sessions	WHOQOL-BREF (WHO Quality of Life-BREF)	Placebo	Germany
Belgacem *et al.* [21] (2013)	33/34	56.6 (20.4)/ 62.5 (18.1)	M-40.6%, F- 59.4%/ M-42.4%,F-57.6%	Caregiver Educational Program	SF36 Health Survey-36 items	Placebo	France
Bahrami and Farzi [26] (2014)	32/32	36.94 (11.3)/ 38.97 (10.2)	M-43.8%, F-56.2%/ M-28.1%, F-71.9%	Supportive educational program based on the COPE model	(CQOL-C) Caregiver Quality of Life Index-Cancer	Placebo	Iran
Mabel Leow *et al.* [28] (2015)	40/40	NA	NA	CCP (Caring for Caregiver Programme)	EORTC Quality of life (QOL)	Placebo	Singapore
Susan C. McMillan *et al.* [24] (2006)	111/109	63.06 (13.58)/ 59.98 (15.27)	M-24%, F-76%/ M-20%, F-80%	Coping skills intervention	(CQOL-C) Caregiver Quality of Life Index-Cancer-35 items	Placebo	South Florida
Joanne M. Shaw *et al.* [29] (2015)	64/64	55.7 (14.9)/ 52.7 (11.8)	M-27%, F-73%/ M-28%, F-72%	Family Connect intervention	Caregiver QOL Short Form (SF)-12 items	Placebo	Australia
Virginia Sun *et al.* [25] (2015)	197/157	57.54 (14.31)/ 57.23 (13.16)	M-39.4%, F-60.6% /M-36.2%, F-63.8%	Palliative care intervention	Caregiver QOL tool	Placebo	California
Lapid, M. I. *et al.* [27] (2016)	47/53	Mean age & SD= NA	Male and Female= NA	QOL intervention	(CQOL-C) Caregiver Quality of Life Index-Cancer-35 items	Placebo	Rochester, USA

**Note: **EG- Experimental Group, CG- Control Group

## Data Availability

Access to the data will be provided to any researcher who has any queries or in case the data are needed for further study. The data will be shared on request from the corresponding author [K.R].

## LIST OF ABBREVIATION

PRISMA: Preferred Reporting Items for Systematic Reviews and Meta-Analyses
IARC: International Agency for Research on Cancer
QOL: Quality of Life
RCT: Randomised controlled trial
MD: Mean Differences

## STANDARDS OF REPORTING

PRISMA methodology and guidelines were followed.

## SUPPLEMENTARY MATERIAL

PRISMA checklist is available as supplementary material on the publisher’s website along with the published article.
